# Supplementary material for: A Likelihood Approach for Real-Time Calibration of Stochastic Compartmental Epidemic Models
Source: PLoS Comput Biol. 2017 Jan 17;13(1):e1005257. doi: 10.1371/journal.pcbi.1005257 (PMC5240920; doi:10.1371/journal.pcbi.1005257)
Supplement: S1 File — (TAR.GZ) [file pcbi.1005257.s014.tar.gz › HSPH_Online-SI-Revision/EnKF07/n100-severe/n100-severe_EnKF2_table-4.pdf]

1	newly 3 weeks spec	6483.	954.46	{2., 792.}	95.0554	1.75435	newly 3 weeks cumul	16 417.	12 286.2	{1948., 18 420.}	118.105	0.736132	newly 5 weeks spec	6768.	221.87	{0., 225.}	98.316	2.71939	newly 5 weeks cumul	30 019.	12 967.	{1965., 19 213.}	102.258	0.926709	newly 54 weeks cumul	56 585.	13 171.9	{1965., 19 315.}	92.8393	1.145
2	newly 3 weeks spec	9252.	238.76	{0., 550.}	97.4194	2.26894	newly 3 weeks cumul	20 576.	6986.09	{1151., 17 754.}	73.6677	0.696157	newly 5 weeks spec	7700.	45.48	{0., 94.}	99.4094	3.19902	newly 5 weeks cumul	37 668.	7131.77	{1198., 17 759.}	83.4092	0.932579	newly 61 weeks cumul	50 118.	7192.25	{1206., 17 759.}	86.611	1.04795
3	newly 3 weeks spec	13 618.	1320.47	{237., 2980.}	90.3035	1.25525	newly 3 weeks cumul	28 056.	27 481.	{8667., 67 660.}	82.898	0.413921	newly 5 weeks spec	10 200.	255.15	{5., 229.}	97.4985	2.402	newly 5 weeks cumul	51 910.	28 241.2	{8818., 67 957.}	69.3839	0.542616	newly 62 weeks cumul	64 948.	28 636.9	{8829., 67 962.}	68.6816	0.602249
4	newly 3 weeks spec	8496.	2722.76	{0., 5096.}	105.644	2.30188	newly 3 weeks cumul	19 445.	8666.98	{898., 45 465.}	91.2663	0.938939	newly 5 weeks spec	8732.	1067.01	{0., 8696.}	89.1966	2.9005	newly 5 weeks cumul	37 352.	11 771.4	{898., 50 494.}	89.9234	1.14244	newly 60 weeks cumul	61 359.	12 930.4	{898., 61 133.}	85.4775	1.29912
5	newly 3 weeks spec	5838.	1017.49	{1., 1152.}	95.0701	1.76047	newly 3 weeks cumul	15 689.	10 883.5	{2000., 8621.}	108.535	0.682972	newly 5 weeks spec	5980.	232.71	{0., 246.}	98.1216	2.5843	newly 5 weeks cumul	27 938.	11 606.2	{2000., 9504.}	98.112	0.866569	newly 54 weeks cumul	56 535.	11 817.3	{2000., 9782.}	91.0245	1.11497
6	newly 3 weeks spec	8007.	2455.29	{0., 3996.}	105.544	2.15258	newly 3 weeks cumul	17 018.	8223.71	{903., 27 064.}	93.4033	0.882857	newly 5 weeks spec	9118.	986.66	{0., 399.}	89.8394	2.89625	newly 5 weeks cumul	35 492.	11 044.3	{917., 29 082.}	90.255	1.11237	newly 60 weeks cumul	63 071.	12 186.9	{917., 51 188.}	85.9047	1.29908
7	newly 3 weeks spec	9834.	263.29	{0., 542.}	97.3227	2.40258	newly 3 weeks cumul	19 745.	6323.63	{1026., 14 857.}	76.3033	0.746598	newly 5 weeks spec	11 408.	72.73	{0., 94.}	99.3625	3.30621	newly 5 weeks cumul	43 078.	6523.35	{1118., 14 865.}	86.4384	1.05188	newly 61 weeks cumul	66 246.	6674.83	{1143., 15 082.}	90.1757	1.22628
8	newly 3 weeks spec	6637.	1324.05	{248., 1131.}	91.5876	1.11378	newly 3 weeks cumul	16 097.	6239.64	{1163., 4323.}	98.0606	0.848496	newly 5 weeks spec	7091.	572.75	{74., 787.}	91.9229	1.48339	newly 5 weeks cumul	30 302.	7636.5	{1579., 5772.}	92.7242	0.992583	newly 57 weeks cumul	53 290.	10 765.4	{2024., 12 558.}	88.702	1.09494
9	newly 3 weeks spec	10 162.	1099.91	{253., 1795.}	89.1762	1.17757	newly 3 weeks cumul	16 274.	25 688.8	{5678., 68 120.}	111.778	0.328148	newly 5 weeks spec	13 814.	146.82	{4., 247.}	98.9372	2.63459	newly 5 weeks cumul	44 791.	26 170.7	{6869., 68 524.}	71.6945	0.520296	newly 62 weeks cumul	61 547.	26 661.6	{8629., 68 533.}	68.9141	0.594053
10	newly 3 weeks spec	8608.	721.94	{0., 1038.}	96.1197	2.99211	newly 3 weeks cumul	19 464.	10 250.1	{484., 12 479.}	79.7718	0.725358	newly 5 weeks spec	8668.	170.67	{0., 535.}	98.031	3.30107	newly 5 weeks cumul	37 387.	10 735.4	{534., 12 568.}	83.4477	0.931024	newly 55 weeks cumul	57 915.	11 337.5	{541., 12 793.}	84.7941	1.04691
11	newly 3 weeks spec	9885.	265.99	{0., 538.}	97.3092	2.33976	newly 3 weeks cumul	20 715.	6852.25	{1083., 17 770.}	74.5044	0.723556	newly 5 weeks spec	9915.	70.83	{0., 93.}	99.2856	3.36227	newly 5 weeks cumul	41 751.	7050.33	{1155., 17 798.}	84.8604	0.99864	newly 61 weeks cumul	59 863.	7193.14	{1171., 17 798.}	88.4786	1.14484
12	newly 3 weeks spec	6820.	851.72	{2., 638.}	95.5484	1.80921	newly 3 weeks cumul	17 074.	11 317.7	{1643., 9247.}	112.638	0.752408	newly 5 weeks spec	6925.	213.11	{0., 221.}	98.4264	2.76139	newly 5 weeks cumul	31 324.	11 958.4	{1643., 9364.}	100.071	0.949931	newly 54 weeks cumul	59 414.	12 165.	{1643., 9371.}	92.5602	1.17597
13	newly 3 weeks spec	6696.	972.78	{2., 753.}	94.9654	1.73377	newly 3 weeks cumul	17 080.	12 376.1	{2059., 18 552.}	116.231	0.737985	newly 5 weeks spec	6771.	227.14	{0., 252.}	98.2336	2.69887	newly 5 weeks cumul	30 784.	13 072.8	{2116., 19 323.}	101.545	0.923421	newly 54 weeks cumul	57 756.	13 283.	{2116., 19 391.}	92.496	1.13959
14	newly 3 weeks spec	5021.	997.6	{1., 923.}	96.3888	1.76338	newly 3 weeks cumul	12 730.	10 519.3	{1392., 8885.}	120.388	0.675927	newly 5 weeks spec	5391.	214.63	{0., 240.}	98.4806	2.66061	newly 5 weeks cumul	23 614.	11 189.9	{1392., 8996.}	104.096	0.876143	newly 54 weeks cumul	54 929.	11 384.6	{1392., 9002.}	92.7514	1.17764
15	newly 3 weeks spec	6167.	1670.15	{16., 1595.}	91.4459	1.29774	newly 3 weeks cumul	14 161.	10 988.2	{231., 10 459.}	93.6223	0.660918	newly 5 weeks spec	6966.	642.46	{0., 981.}	90.7772	2.23018	newly 5 weeks cumul	28 290.	12 643.1	{238., 11 064.}	90.7199	0.882453	newly 56 weeks cumul	48 068.	14 170.5	{238., 11 282.}	89.3114	1.05826
16	newly 3 weeks spec	5400.	688.28	{0., 1051.}	95.63	2.81211	newly 3 weeks cumul	13 040.	10 180.2	{858., 13 323.}	70.1808	0.471176	newly 5 weeks spec	6295.	321.41	{0., 534.}	96.8132	3.13324	newly 5 weeks cumul	25 735.	10 937.6	{994., 13 399.}	77.843	0.680084	newly 55 weeks cumul	58 209.	12 335.8	{1002., 13 591.}	82.9656	0.933388
17	newly 3 weeks spec	7444.	504.49	{0., 547.}	96.2229	2.3256	newly 3 weeks cumul	14 892.	8250.67	{1015., 15 661.}	81.3284	0.632681	newly 5 weeks spec	8949.	154.38	{0., 98.}	98.2749	3.28744	newly 5 weeks cumul	32 852.	8677.36	{1127., 17 912.}	84.3633	0.911124	newly 61 weeks cumul	50 701.	8937.11	{1130., 17 913.}	86.4116	1.07314
18	newly 3 weeks spec	7109.	1311.71	{85., 2197.}	93.5326	1.42715	newly 3 weeks cumul	17 024.	6680.12	{1283., 7511.}	98.0885	0.951906	newly 5 weeks spec	7086.	353.6	{6., 979.}	95.0099	2.09354	newly 5 weeks cumul	31 968.	7698.56	{1422., 10 569.}	92.182	1.13853	newly 59 weeks cumul	50 495.	8440.27	{1477., 13 459.}	89.8099	1.28647
19	newly 3 weeks spec	9821.	1347.46	{7., 1830.}	92.7517	1.81592	newly 3 weeks cumul	23 696.	6595.34	{52., 6654.}	96.2497	1.34218	newly 5 weeks spec	7954.	416.4	{1., 1110.}	95.2167	2.42437	newly 5 weeks cumul	41 504.	7741.71	{60., 9293.}	92.415	1.50914	newly 59 weeks cumul	56 218.	8612.73	{63., 12 720.}	90.7023	1.59891
20	newly 3 weeks spec	8828.	1237.74	{121., 1167.}	92.2873	1.31274	newly 3 weeks cumul	20 393.	5965.29	{890., 4566.}	97.3573	1.0117	newly 5 weeks spec	8356.	525.69	{42., 655.}	93.7088	1.64225	newly 5 weeks cumul	37 963.	7245.59	{1129., 6082.}	93.4728	1.15543	newly 57 weeks cumul	56 638.	10 299.4	{1292., 7740.}	89.5125	1.19124
21	newly 3 weeks spec	9444.	259.25	{0., 539.}	97.2549	2.3321	newly 3 weeks cumul	18 625.	6453.33	{1000., 15 302.}	74.61	0.717119	newly 5 weeks spec	10 527.	74.84	{0., 101.}	99.2891	3.27486	newly 5 weeks cumul	40 402.	6655.	{1092., 15 302.}	85.4875	1.01944	newly 61 weeks cumul	60 507.	6811.48	{1150., 15 302.}	89.2161	1.18161
22	newly 3 weeks spec	7683.	1769.93	{149., 4976.}	94.4854	1.31863	newly 3 weeks cumul	14 543.	8842.05	{1323., 13 435.}	109.54	0.794599	newly 5 weeks spec	9422.	465.88	{11., 1259.}	95.0554	2.01865	newly 5 weeks cumul	33 493.	10 199.1	{1470., 26 612.}	94.5005	1.04233	newly 59 weeks cumul	53 168.	11 058.1	{1522., 42 534.}	89.6898	1.18053
23	newly 3 weeks spec	16 661.	2530.94	{1., 8229.}	86.0617	1.98344	newly 3 weeks cumul	28 675.	33 768.	{4914., 74 655.}	73.3378	0.368809	newly 5 weeks spec	10 882.	964.58	{0., 3154.}	91.7195	2.86558	newly 5 weeks cumul	56 693.	36 170.9	{6097., 74 655.}	61.1582	0.485926	newly 63 weeks cumul	64 940.	38 929.8	{7213., 79 828.}	59.4805	0.498143
24	newly 3 weeks spec	11 463.	7312.9	{892., 20 704.}	68.4308	0.525175	newly 3 weeks cumul	20 345.	44 555.	{12 644., 93 928.}	139.726	0.330786	newly 5 weeks spec	12 907.	2046.85	{58., 3730.}	84.1416	1.22459	newly 5 weeks cumul	47 801.	50 370.7	{13 546., 104 928.}	67.1915	0.318135	newly 64 weeks cumul	66 552.	53 481.5	{13 620., 109 935.}	58.1729	0.350898
25	newly 3 weeks spec	11 140.	7310.03	{894., 20 604.}	68.5272	0.515089	newly 3 weeks cumul	19 297.	44 402.2	{12 693., 93 992.}	148.864	0.337691	newly 5 weeks spec	11 133.	2065.67	{66., 3768.}	81.7788	1.16698	newly 5 weeks cumul	44 204.	50 259.6	{13 617., 104 708.}	70.2616	0.30826	newly 64 weeks cumul	57 475.	53 412.9	{13 709., 109 697.}	61.0708	0.325451
26	newly 3 weeks spec	9973.	845.98	{19., 1405.}	91.5173	1.5258	newly 3 weeks cumul	21 090.	6021.08	{393., 12 446.}	83.8904	0.941244	newly 5 weeks spec	10 139.	353.13	{2., 614.}	96.5171	2.25029	newly 5 weeks cumul	42 295.	6905.01	{493., 15 493.}	85.0427	1.14346	newly 58 weeks cumul	65 347.	7945.84	{631., 16 944.}	88.4472	1.27309
27	newly 3 weeks spec	9435.	1342.49	{118., 2165.}	91.5836	1.48848	newly 3 weeks cumul	19 821.	6841.49	{1297., 7485.}	95.9672	0.972562	newly 5 weeks spec	9500.	367.19	{5., 822.}	96.1348	2.13948	newly 5 weeks cumul	40 049.	7891.38	{1444., 10 451.}	90.6886	1.18531	newly 59 weeks cumul	58 495.	8637.43	{1506., 13 614.}	88.9257	1.30044
28	newly 3 weeks spec	6339.	734.98	{15., 896.}	88.5449	1.55321	newly 3 weeks cumul	16 315.	4871.11	{221., 9700.}	87.9341	1.06328	newly 5 weeks spec	5894.	313.46	{0., 543.}	95.4214	2.31037	newly 5 weeks cumul	28 643.	5653.96	{264., 10 719.}	86.3761	1.20952	newly 58 weeks cumul	47 081.	6523.47	{291., 11 000.}	88.4823	1.35544
29	newly 3 weeks spec	9036.	1153.09	{7., 1274.}	92.3374	1.66969	newly 3 weeks cumul	21 261.	5930.67	{59., 4167.}	94.7979	1.2279	newly 5 weeks spec	8506.	337.71	{2., 672.}	96.0297	2.29618	newly 5 weeks cumul	39 366.	6868.72	{66., 5619.}	91.3497	1.41541	newly 59 weeks cumul	60 373.	7617.08	{69., 10 346.}	89.9566	1.55335
30	newly 3 weeks spec	6346.	712.14	{0., 1045.}	96.919	2.91159	newly 3 weeks cumul	15 783.	10 367.6	{877., 12 207.}	77.9187	0.576938	newly 5 weeks spec	6273.	170.22	{0., 514.}	97.2865	3.19368	newly 5 weeks cumul	28 864.	10 854.3	{912., 12 280.}	81.2224	0.760732	newly 55 weeks cumul	48 852.	11 474.7	{917., 12 280.}	83.2062	0.911581
31	newly 3 weeks spec	15 806.	7283.8	{878., 20 802.}	67.5301	0.619199	newly 3 weeks cumul	27 617.	44 286.3	{12 666., 93 872.}	97.6749	0.30703	newly 5 weeks spec	9080.	2041.5	{67., 3756.}	79.1978	1.07745	newly 5 weeks cumul	52 596.	50 077.1	{13 138., 104 809.}	64.9226	0.340444	newly 64 weeks cumul	59 587.	53 170.5	{13 686., 109 916.}	60.6577	0.338023
32	newly 3 weeks spec	10 271.	1100.98	{245., 1713.}	89.2807	1.14166	newly 3 weeks cumul	19 060.	21 129.2	{6899., 50 608.}	75.2973	0.316901	newly 5 weeks spec	11 191.	187.46	{5., 233.}	98.3249	2.42735	newly 5 weeks cumul	42 799.	21 704.5	{8864., 50 747.}	65.3488	0.501395	newly 62 weeks cumul	60 379.	22 094.2	{8885., 50 747.}	68.3916	0.608457
33	newly 3 weeks spec	12 313.	1328.01	{225., 2832.}	89.2146	1.21127	newly 3 weeks cumul	26 701.	27 522.4	{8709., 67 556.}	84.9167	0.403268	newly 5 weeks spec	10 094.	255.48	{4., 234.}	97.469	2.39815	newly 5 weeks cumul	49 375.	28 288.4	{8845., 67 807.}	69.4725	0.527027	newly 62 weeks cumul	64 967.	28 632.5	{8853., 67 817.}	68.6765	0.602207
34	newly 3 weeks spec	7987.	1014.95	{3., 1074.}	97.2534	1.90555	newly 3 weeks cumul	19 140.	17 548.2	{1285., 78 755.}	131.109	0.807662	newly 5 weeks spec	7767.	209.99	{0., 228.}	98.2334	3.00682	newly 5 weeks cumul	35 440.	18 215.8	{1285., 83 448.}	105.598	0.987093	newly 54 weeks cumul	57 950.	18 405.8	{1285., 85 268.}	93.4156	1.13905
35	newly 3 weeks spec	12 123.	3221.66	{3., 8339.}	79.4594	1.7448	newly 3 weeks cumul	20 957.	40 735.6	{7229., 92 001.}	128.499	0.399795	newly 5 weeks spec	11 360.	1001.38	{0., 3227.}	91.5595	2.83365	newly 5 weeks cumul	47 195.	43 390.6	{7451., 107 389.}	63.3701	0.393169	newly 63 weeks cumul	60 555.	45 940.9	{7549., 114 890.}	58.6023	0.423473
36	newly 3 weeks spec	8896.	491.74	{0., 542.}	96.2122	2.40617	newly 3 weeks cumul	16 239.	8494.71	{1009., 18 084.}	80.0524	0.642011	newly 5 weeks spec	12 338.	153.31	{0., 104.}	98.7574	3.44309	newly 5 weeks cumul	40 963.	8919.87	{1066., 18 223.}	84.9029	0.9772	newly 61 weeks cumul	64 577.	9173.59	{1096., 18 223.}	87.2538	1.15071
37	newly 3 weeks spec	11 908.	2371.78	{1., 8031.}	82.9125	2.01899	newly 3 weeks cumul	22 407.	34 406.5	{4895., 73 794.}	95.1968	0.357573	newly 5 weeks spec	9251.	928.32	{0., 3008.}	91.3897	2.98188	newly 5 weeks cumul	44 615.	36 713.8	{6100., 73 794.}	58.5316	0.390856	newly 63 weeks cumul	54 448.	39 090.2	{7182., 74 547.}	56.7984	0.420324
38	newly 3 weeks spec	11 993.	267.37	{0., 555.}	97.7706	2.45612	newly 3 weeks cumul	24 507.	6850.19	{1013., 17 935.}	77.8461	0.800423	newly 5 weeks spec	11 210.	72.58	{0., 97.}	99.3525	3.38604	newly 5 weeks cumul	48 530.	7051.01	{1130., 17 946.}	86.5403	1.06811	newly 61 weeks cumul	66 756.	7193.72	{1166., 17 947.}	89.4553	1.19732
39	newly 3 weeks spec	6786.	773.63	{11., 1016.}	88.6421	1.58238	newly 3 weeks cumul	16 864.	5011.57	{207., 9793.}	87.4179	1.08175	newly 5 weeks spec	6041.	339.74	{0., 575.}	95.1038	2.2991	newly 5 weeks cumul	29 945.	5844.64	{249., 10 801.}	85.9398	1.23337	newly 58 weeks cumul	46 740.	6805.56	{270., 11 073.}	87.9464	1.36115
40	newly 3 weeks spec	7110.	267.61	{0., 535.}	96.2361	2.23975	newly 3 weeks cumul	16 261.	6861.56	{1059., 17 740.}	70.633	0.626204	newly 5 weeks spec	7170.	70.51	{0., 93.}	99.0166	3.18852	newly 5 weeks cumul	31 380.	7059.75	{1160., 17 758.}	81.1301	0.874518	newly 61 weeks cumul	47 800.	7199.17	{1174., 17 758.}	86.0641	1.0446
41	newly 3 weeks spec	12 763.	2128.24	{0., 6147.}	86.3092	2.15272	newly 3 weeks cumul	25 803.	33 947.4	{5014., 72 781.}	76.533	0.324192	newly 5 weeks spec	9905.	797.94	{0., 2984.}	92.2859	3.04203	newly 5 weeks cumul	48 570.	35 967.1	{7151., 72 781.}	56.2639	0.38776	newly 63 weeks cumul	62 184.	37 994.1	{7690., 72 781.}	55.1542	0.429565
42	newly 3 weeks spec	7600.	1453.01	{48., 1575.}	91.4036	1.39054	newly 3 weeks cumul	17 610.	9902.	{567., 10 332.}	91.123	0.74452	newly 5 weeks spec	7814.	513.03	{1., 980.}	93.4345	2.31875	newly 5 weeks cumul	33 674.	11 259.5	{614., 10 872.}	90.2999	0.955946	newly 56 weeks cumul	56 523.	12 783.4	{623., 11 298.}	89.1366	1.12435
43	newly 3 weeks spec	5514.	704.65	{0., 1021.}	95.7398	2.80229	newly 3 weeks cumul	14 068.	9721.41	{1062., 12 141.}	71.5015	0.501132	newly 5 weeks spec	5382.	321.17	{0., 565.}	96.623	3.05438	newly 5 weeks cumul	25 003.	10 485.8	{1154., 12 170.}	77.8743	0.671966	newly 55 weeks cumul	42 797.	11 906.4	{1164., 13 237.}	80.7532	0.81315
44	newly 3 weeks spec	9897.	1106.94	{247., 1693.}	88.8154	1.12295	newly 3 weeks cumul	17 922.	21 134.1	{7067., 50 420.}	76.7272	0.305066	newly 5 weeks spec	12 427.	194.04	{5., 253.}	98.4386	2.43957	newly 5 weeks cumul	43 675.	21 725.	{8885., 50 604.}	65.4474	0.508982	newly 62 weeks cumul	65 197.	22 101.1	{8973., 50 605.}	69.3673	0.637226
45	newly 3 weeks spec	7988.	2141.05	{0., 1702.}	105.107	2.21677	newly 3 weeks cumul	17 198.	7666.88	{909., 26 814.}	93.1485	0.915383	newly 5 weeks spec	9497.	794.55	{0., 295.}	91.7093	3.00434	newly 5 weeks cumul	36 455.	10 025.7	{911., 27 518.}	90.6909	1.15835	newly 60 weeks cumul	60 722.	10 739.3	{911., 27 568.}	87.7501	1.33017
46	newly 3 weeks spec	8753.	1353.62	{136., 2181.}	92.0893	1.40223	newly 3 weeks cumul	17 795.	6954.49	{1317., 9630.}	96.8922	0.873713	newly 5 weeks spec	10 647.	361.67	{10., 823.}	96.6031	2.17206	newly 5 weeks cumul	39 229.	8000.7	{1465., 10 677.}	90.6871	1.12098	newly 59 weeks cumul	63 639.	8725.64	{1516., 14 226.}	88.6037	1.2777
47	newly 3 weeks spec	6293.	673.07	{0., 1093.}	94.836	2.78443	newly 3 weeks cumul	16 455.	10 126.7	{1586., 13 525.}	72.4719	0.505891	newly 5 weeks spec	6515.	314.7	{0., 583.}	96.9863	3.11211	newly 5 weeks cumul	29 490.	10 870.5	{1727., 13 556.}	78.2295	0.677482	newly 55 weeks cumul	54 744.	12 191.2	{1837., 13 798.}	83.3538	0.852467
48	newly 3 weeks spec	8060.	2928.62	{0., 5798.}	107.733	2.20444	newly 3 weeks cumul	18 107.	9047.5	{911., 45 541.}	95.0831	0.926823	newly 5 weeks spec	8793.	1144.06	{0., 8685.}	88.118	2.85291	newly 5 weeks cumul	35 919.	12 356.	{913., 74 282.}	91.3469	1.13539	newly 60 weeks cumul	61 312.	13 637.7	{913., 80 533.}	85.3764	1.30127
49	newly 3 weeks spec	13 841.	6325.87	{947., 20 482.}	65.9544	0.62231	newly 3 weeks cumul	23 044.	43 703.3	{12 213., 95 942.}	118.474	0.316108	newly 5 weeks spec	12 770.	1585.92	{79., 3606.}	87.5809	1.27944	newly 5 weeks cumul	52 454.	48 346.1	{13 533., 104 995.}	63.8568	0.343489	newly 64 weeks cumul	65 680.	50 860.1	{14 065., 107 163.}	57.0873	0.353929
50	newly 3 weeks spec	12 684.	2097.	{0., 6067.}	86.4991	2.18818	newly 3 weeks cumul	25 589.	33 633.3	{4963., 72 755.}	78.4768	0.344289	newly 5 weeks spec	9733.	793.67	{0., 2928.}	92.2015	3.07739	newly 5 weeks cumul	48 457.	35 645.	{6077., 72 755.}	56.8727	0.406797	newly 63 weeks cumul	60 105.	37 671.2	{7188., 72 755.}	55.5049	0.440632
